# Development of a Novel Fluorophore for Real-Time Biomonitoring System

**DOI:** 10.1371/journal.pone.0048459

**Published:** 2012-11-02

**Authors:** Hyun-Ok Song, Binna Lee, Ram Prasad Bhusal, Byounghun Park, Kyoungsik Yu, Chom-Kyu Chong, PyoYun Cho, Sung Yeon Kim, Hak Sung Kim, Hyun Park

**Affiliations:** 1 Zoonosis Research Center, Department of Infection Biology, Wonkwang University College of Medicine, Iksan, Jeonbuk, Republic of Korea; 2 College of Pharmacy, Institute of Pharmaceutical Research and Development, Wonkwang University, Iksan, Jeonbuk, Republic of Korea; 3 Department of Electrical Engineering, Korea Advanced Institute of Science and Technology, Daejeon, Republic of Korea; 4 Department of Biochemistry, Division of Life Science, Chungbuk National University, Cheongju, Chungbuk, Republic of Korea; Centro de Pesquisa Rene Rachou/Fundação Oswaldo Cruz (Fiocruz-Minas), Brazil

## Abstract

Rapid in-field diagnosis is very important to prevent the outbreak of various infectious and contagious diseases. Highly sensitive and quantitative detection of diseases can be performed using fluorescent immunochemical assay with specific antigen-antibody binding and a good quality fluorophore. This can lead to the development of a small, portable, quantitative biosensor to transmit diagnostic results to a control center in order to systematically prevent disease outbreaks. In this study, we developed a novel fluorophore, coumarin-derived dendrimer, with high emission intensity, strong signal brightness, and high photostability. It is easily coupled with biomolecules and emits strong and stable fluorescence at 590 nm with excitation at 455 nm. Application to fluorescent immunochromatographic test (FICT) showed that the novel coumarin-derived dendrimer bioconjugate could detect antigens at amount as low as 0.1 ng. The clinical results and the spectral characteristics of the novel coumarin-derived dendrimer open, for the first time, the possibility of developing a cost/energy efficient LED-based portable quantitative biosensor for point-of-care (POC) disease diagnosis, which can permit real time monitoring (U-healthcare system) by a disease control center.

## Introduction

Accurate and prompt diagnosis is one of the keys for effective disease management. Rapid diagnosis is especially important for infectious diseases such as swine flu and avian flu to prevent their spread. Many people worldwide have suffered and some have died from the novel influenza A virus, pandemic influenza A/H1N1 2009 (referred to as swine flu) [1], [2], and avian influenza, A/H5N1 [3], [4] because the influenza virus is very contagious and can cause a life-threatening illness. In order to prevent outbreaks of many infectious diseases, including influenza, AIDS, and malaria, it is necessary to develop a centrally managed real-time monitoring system via the use of precise and sensitive diagnostic instruments, in particular point-of-care (POC) or field-deployable portable biosensors [5]–[7]. The biosensors should present quantitative diagnostic results and conveniently transport the results to the control center to systematically manage disease prevention. Therefore, recent progress in developing fluorescent diagnostic devices is highly innovative and important for the development of sensitive biosensors and their clinical application [6]–[8].

Fluorescence techniques have been used for various analytical purposes in biological and biomedical research, and clinical diagnosis [9]–[11] because fluorescent detection is one of the most sensitive methods for identifying organic and/or inorganic compounds even in low-concentration analytes. PCR or real-time PCR using fluorescent dyes is considered to be the most sensitive and accurate method to detect infections by influenza virus [2], [12]–[15] and malaria parasite [16]–[20]. However, the application of real-time PCR in field diagnosis has some limitations including relatively longer reaction time (1–5 h) to detect, heavier and bigger machine body making difficult to carry, and need for electric power to operate. Therefore, rapid diagnostic test (RDT) still has advantages in field diagnosis because it is the easiest and simplest way to diagnose many infectious diseases within a short time (15–30 min). Fluorescent immunorchromatographic test (FICT) is one of the formats of RDT. Beyond the advantages as a RDT, it can allow us to diagnose infections quantitatively, and thereby to transform the diagnostic results to numerical digits that can be transported to monitoring center via portable biosensor. To perform FICT, biomolecules such as an antibody and an antigen can be tagged with a fluorescent chemical group by a simple chemical reaction and the fluorescent bioconjugates enable sensitive and quantitative detection of molecules such as DNA and protein. Many organic and/or inorganic fluorescent dye labels [21]–[24] have been developed to fulfill ideal label requirements, including cost, stability, and sensitivity [25]. However, the traditional fluorophores and current fluorescent dyes have limited use because of intrinsic problems that result in low sensitivity and stability, including low emission intensity, interference, rapid photobleaching, and requirement of a high energy source (laser diode) for excitation.

To achieve quantitative and sensitive fluorescent detection in biosensors, fluorescent dyes should be improved by increasing stability and intensity. Quantum dots (QD) are inorganic nanocrystals that are an emerging candidate for the ideal fluorescent label for biomolecules. They have unique properties, such as size- and composition-tunable light emission from visible (red, orange, yellow, and green) to infrared wavelengths, strong signal intensity, and high photostability (resistance to photobleaching) [8], [26], [27]. In addition, simultaneous multiplex labeling and detection are possible due to the broad excitation spectrum, which enables excitation of distinct QDs at a single wavelength [28]. Recent studies have reported the successful application of QDs in bioassays, *in vitro*/*in vivo* biosensing, and bioimaging [8], [26], [29]–[32], demonstrating that the use of QDs results in tremendous improvement of sensitivity and multiplex labeling compared to organic fluorophores. However, QDs also have specific disadvantages. They have a relatively large size, which might alter the natural behavior of small biomolecules [30], [33]; instability in manufacturing; and quenching in solution due to molecular collision [34]. The relatively tedious process of preparing bioconjugates also diminishes the utility of QDs for bioimaging and bioassays such as fluorescent labeling for cellular imaging, immunohistochemistry, fluorescent immunochromatographic assay, and fluorescence-linked immunosorbent assay [28], [35]–[37].

Recently, an immunochromatographic fluorescent biosensor based on laser diode (LD) induced QD has been described [8]. The authors reported the development of a rapid and quantitative tool to detect trace amounts of chemicals, therefore presenting great promise for the development of in-field, portable, and quantitative biosensors of biomarkers. The LD light source in the biosensor is used in many analytical instruments, including diagnostic devices, because of its good monochromaticity and beam focusing capacity [38]. However, LD is expensive, difficult to operate, and has relatively limited emission wavelength selections and a short lifetime compared to light-emitting diode (LED) sources. LED is considered as a more efficient light source than LD because of its low cost, stable output power with less electrical power input, its long lifetime, and its wider emitting region (390 nm to 750 nm in visible wavelength and infrared wavelength) [38]. These advantages make LED the leading light source for a number of analytical devices. For instance, conventional fluorescence microscopic detection of infectious microorganisms is being improved with LED-based fluorescence microscopy, which allows a reliable detection sensitivity, and cost-effectiveness originating from less power consumption [39]–[43].

Here, we report an LED-based novel organic fluorophore, coumarin-derived dendrimer, which has high emission intensity, strong signal brightness, and high photostability. We further evaluate this novel coumarin-derived dendrimer for its application to the clinical diagnosis of malaria through fluorescence-based immunochemical assays. This novel coumarin-derived dendrimer may promote the development of portable quantitative biosensors for point-of-care (POC) and/or clinical diagnosis of diseases.

### Synthesis of Coumarin-derived Dendrimers

Coumarin (*2H*-chromen-2-one), a benzo-α-pyrone, consists of fused pyrone and benzene rings with a pyrone carbonyl group at position 2, and is well known as fluorescent material because of its high photostability and photoluminescence efficiency [44]–[46]. Coumarins are an important group of organic compounds that are used as additives to food and cosmetics, optical brightening agents, and dispersed fluorescent and laser dyes [47], [48]. A number of 7-aminocoumarin derivatives bearing an aromatic or heteroaromatic radical in the 3-position serve as valuable fluorescent dyes [49].

The most important functional groups of antibodies suitable for derivatization are free amino groups of lysine. Amines on the lysine are reasonably good nucleophiles above pH 8.0 (pK_a_ = 9.18). Therefore, they react easily and cleanly with a variety of electrophilic groups to form stable peptide bonds [50]. NHS ester (*N*-hydroxysuccinimide ester) creates a stable amide with primary or secondary amines [51]. It is generally stable in aqueous media for the period of time necessary for the coupling reaction, and shows good reactivity and selectivity with aliphatic amines [52]. It is important to minimize equivalents of electrophilic organic dyes to avoid reactions with amino groups in the variable region in the antibody, which is the sensitive area for interaction with antigens.

**Figure 1:**
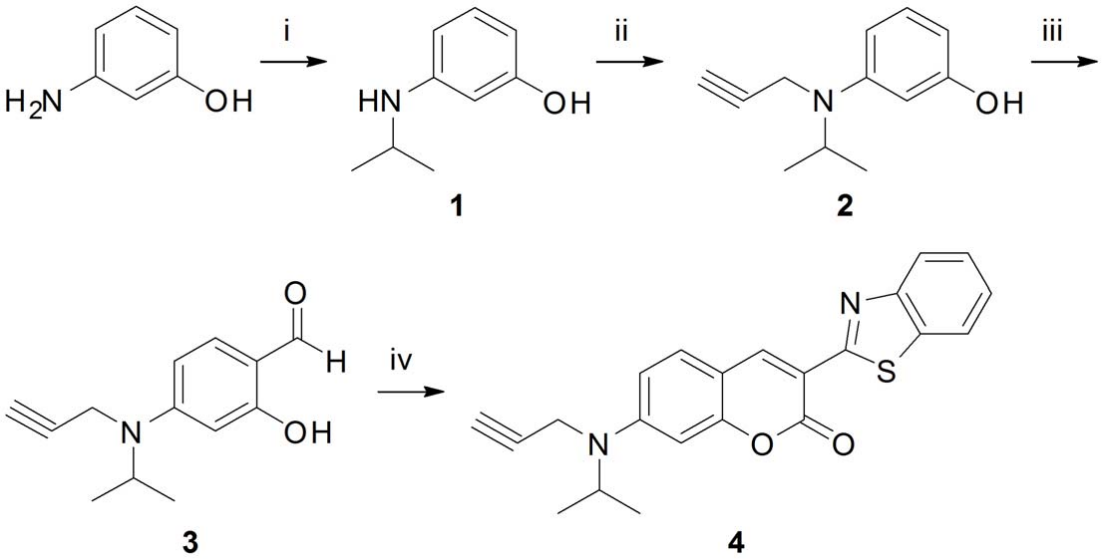
Synthesis of benzothiazolyl coumarin with propargyl group. (i) NaBH(OAc)_3_, acetone, rt, 12 h, 78%. (ii) propargyl chloride, Hunig’s base, toluene, rt, 10 h, 89%. (iii) POCl_3_, DMF, rt, 12 h, 82%. (iv) ethyl 2-(benzo[d]thiazol-2-yl)acetate, piperidine, ethanol, reflux, 2 h, 50%.

The synthesis of the coumarin dendrimer containing succinimide ester started from 3-aminophenol (see Supporting Protocol S1). First, we synthesized benzothiazolyl coumarin **4** containing propargyl group (Figure 1). Reductive amination to 3-aminophenol gave *N*-isopropylaminophenol **1** with 78% yield, followed by the addition of a propargyl group to the amino moiety to give dialkylaniline **2** with 89% yield. Introduction of an aldehyde group was accomplished by Vilsmeier-Haack reaction using DMF and POCl_3_, which afforded 2-hydroxybenzaldehyde **3** with 82% yield [53]. The synthesis of benzothiazolyl coumarin skeleton **4** was carried out according to known protocol [54], [55] by condensation of 2-hydroxybenzaldehyde **3** and ethyl 2-(benzo[d]thiazol-2-yl)acetate in the presence of piperidine in ethanol with 50% yield (Figure 1).

**Figure 2:**
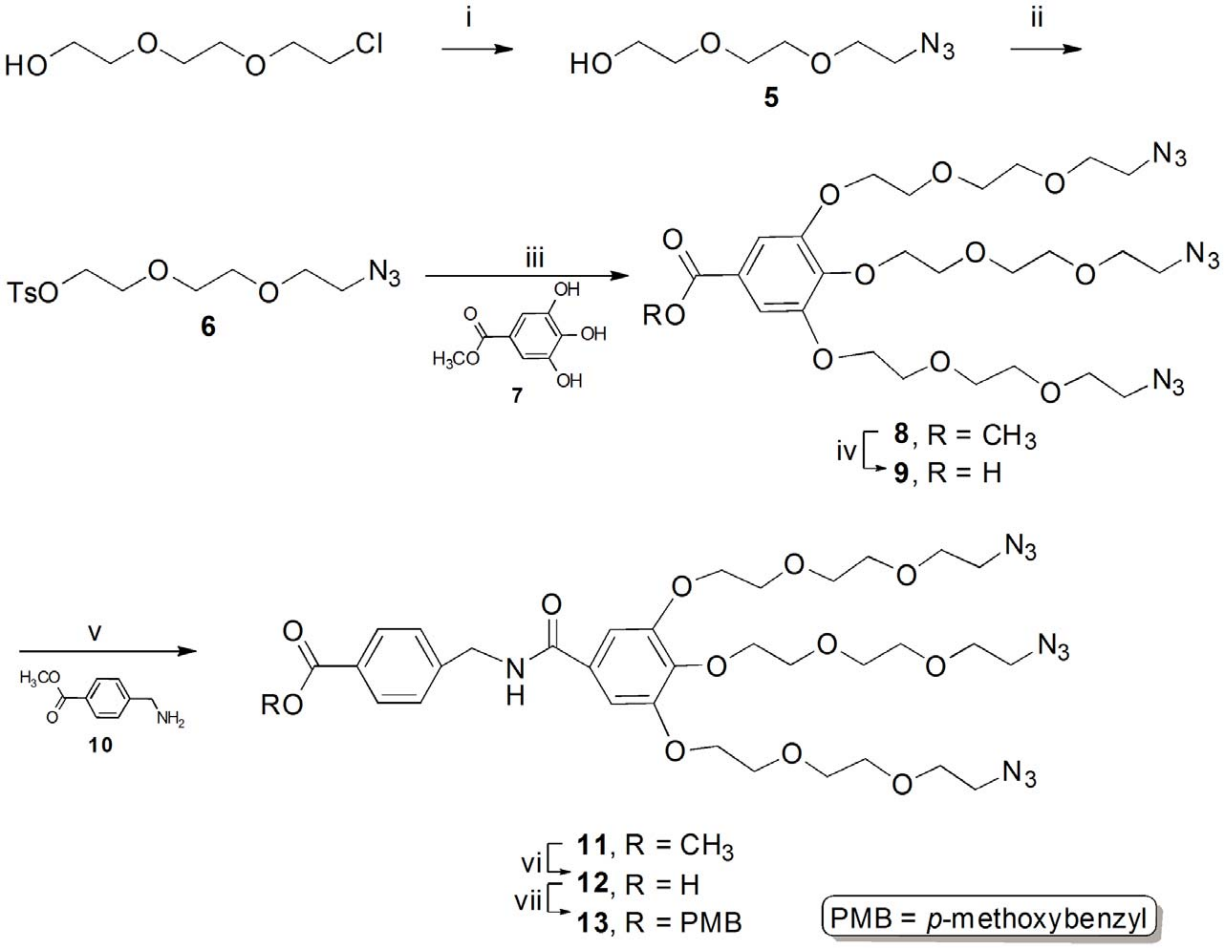
Synthesis of linker with three azido groups. (i) NaN_3_, DMF, 100^o^C, 12 h, 84%. (ii) TsCl, DMAP, TEA, DCM, rt, 12 h, 90%. (iii) K_2_CO_3_, acetone, reflux, 17 h, 94%. (iv) aq. LiOH, THF, reflux, 3 h, 95%. (v) EDAC.HCl, TEA, DCM, rt, 36 h, 60%. (vi) aq. LiOH, THF, rt, 10 h, 99%. (vii) PMB, NaHCO_3_, DMF, rt, 10 h, 82%.

Next, we focused on the synthesis of the gallic acid-derived linker with 3 terminal azido groups and the protected carboxylic acid (Figure 2). We selected triethylene glycol for synthesis of the linker because of its water solubility and easy structural modification [56]. High water solubility of the linker was expected to help in the azide–alkyne Huisgen cycloaddition in the aqueous media. Substitution of azide to chloro group of commercially available triethylene glycol chloride afforded triethylene glycol azide **5** with 84% yield, followed by tosylation to give tosylate **6** with 90% yield. The methyl gallate **7** was prepared with 95% yield by Fischer’s esterification. The base-catalyzed *O*-alkylative coupling of tosylate **6** with methyl gallate **7** afforded the compound **8** with 94% yield. The ester group was readily hydrolyzed by lithium hydroxide to give the corresponding acid **9** with a 95% crude yield. The coupling of the acid **9** and amine **10** [57] in the presence of EDAC and TEA furnished the compound **11** with 60% yield. After base-promoted hydrolysis of the methyl ester of the compound **11** the carboxylic acid group in the compound **12** was protected by a *p*-methoxybenzyl group with 82% yield to yield a three azido groups-terminated dendrimer **13** (Figure 2).

**Figure 3:**
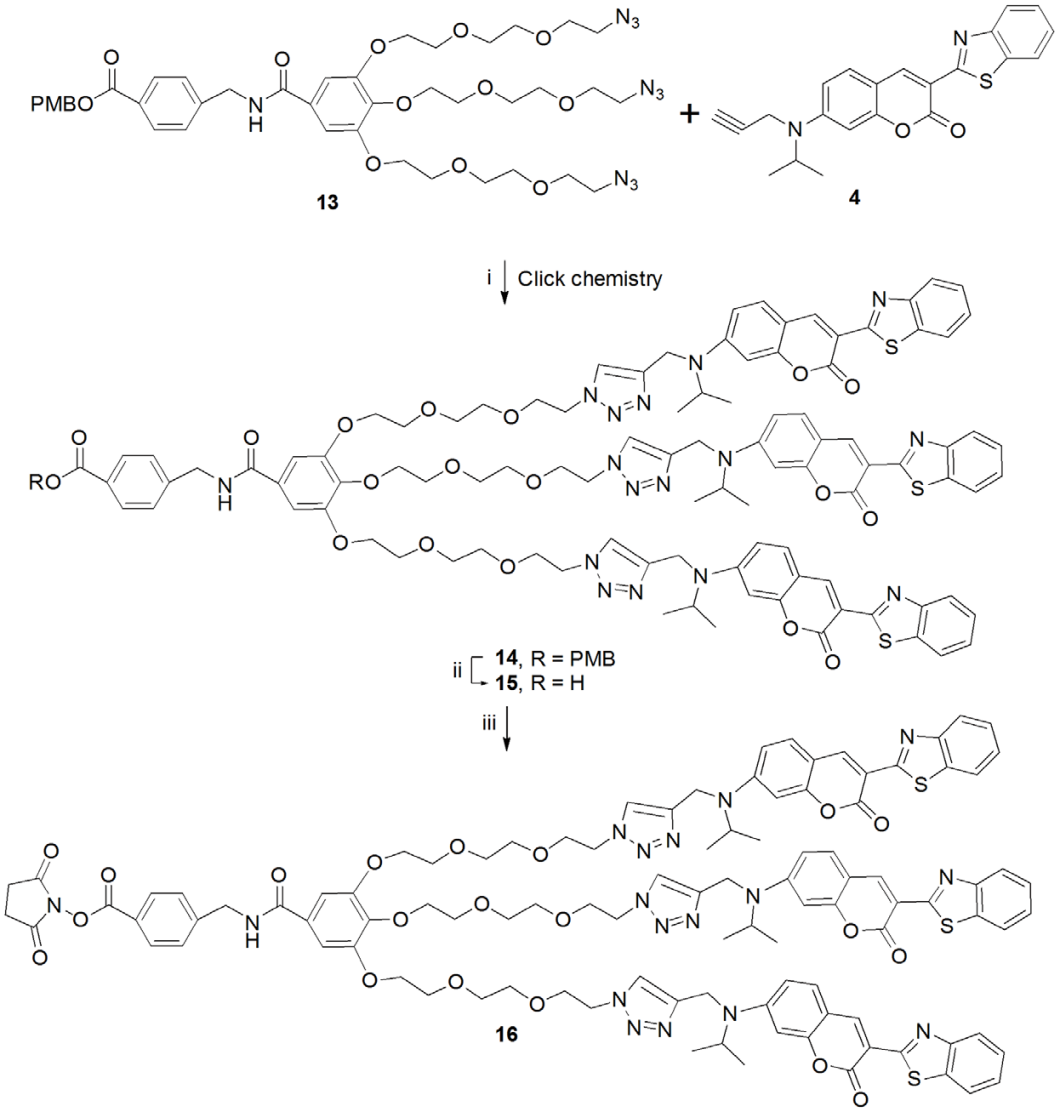
Synthesis of succinimidyl ester of coumarin dendrimer. (i) CuSO_4_.5H_2_O, Na ascorbate, DCM, H_2_0, rt, 8 h, 60%. (ii) TFA, DCM, rt, 36 h, 95%. (iii) NHS, EDAC, DCM, rt, 6 h, 92%.

Coupling of tri-azido gallate **13** with *N*-propargyl benzothiazolyl coumarin **4** was achieved by Click chemistry [58], [59] according to the neutral copper-catalyzed Huisgen 1,3-dipolar cycloaddition of azides and terminal alkynes [60], [61]. Finally, azido linker **13** and *N*-propargyl benzothiazolyl coumarin **4** were coupled using Cu(I) catalyzed [3+2] cycloaddition to give the compound **14** with 60% yield [62]. In the Huisgen 1,3-dipolar cycloaddition, the yield and selectivity were increased when copper sulfate pentahydrate and sodium ascorbate were used as additives. The acid **15** obtained with 95% yield by removing the PMB group was treated with *N*-hydroxysuccinimide in the presence of EDAC to yield the coumarin dendrimer **16** containing the succinimidyl activated ester with 92% yield (Figure 3).

### Spectroscopic Properties of Coumarin-derived Dendrimer

**Figure 4:**
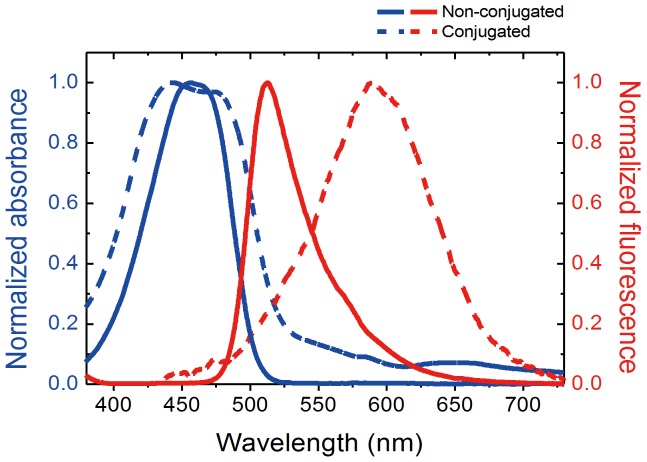
Spectral properties of coumarin-derived dendrimer and bioconjugate. Excitation and emission spectra of coumarin-derived dendrimer are indicated at 455 nm and 512 nm with blue and red solid lines, respectively. The emission spectral shift in bioconjugate of coumarin-derived dendrimer and monoclonal antibody is shown at 590 nm in red dashed line.

**Figure 5:**
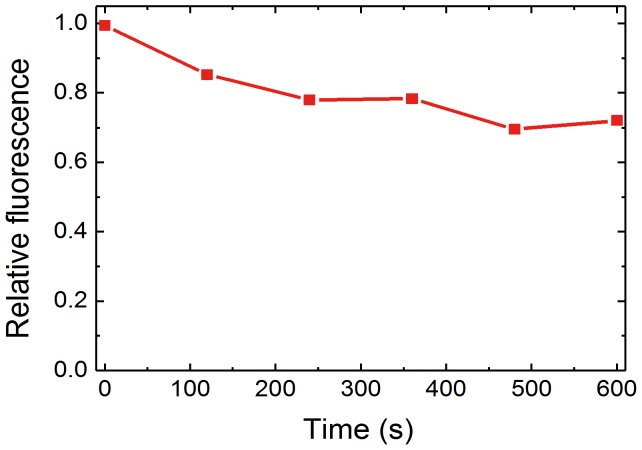
Photostability of bioconjugate of coumarin-derived dendrimer and monoclonal antibody. The mAb bioconjugate of coumarin-derived dendrimer is monitored for photostability. Relative fluorescence intensity drops less than 20% within 600 s.

The spectroscopic properties of the newly synthesized coumarin-derived dendrimer were examined. As shown in Figure 4, the coumarin-derived dendrimer had a relatively wide absorption wavelength range centered on 455 nm, and light sources with broad linewidths, such as light emitting diodes, can thus be employed for fluorescent excitation. After bioconjugation, the peak fluorescent emission wavelength shifted from 520 nm to 590 nm (Figure 4). The redshift of fluorescent emission facilitates the separation of absorption and fluorescence for sensitive and cost-effective detection of fluorophore bioconjugates with decreased noise. It is also worth noting that the fluorescence intensity drops less than 20% within 10 min, indicating excellent photostability of the coumarin-derived dendrimer and mAb bioconjugates (Figure 5). Considering the fluorescence intensity of Alexa dye, one of the brightest and the most photostable organic dyes, drops to 60% within 10 min after its conjugation to antibody [63], [64], the photostability is remarkably improved in bioconjugate of coumarin-derived dendrimer.

### Clinical Application of the Coumarin-derived Dendrimer to Fluorescence-linked Immunosorbent Assay (FLISA) for Detecting Malaria Parasite

The efficacy of coumarin-derived dendrimer for malaria diagnosis, where most organic and/or inorganic fluorescent dyes are used, was investigated. Malaria diagnosis involves the identification of malaria parasites (*Plasmodium* species) or parasite products (antigens) in the blood [65]. The most widely used technique is immunochemical testing, including enzyme-linked immunosorbent assay (ELISA) and immunochromatography (ICT) in the form of lateral-flow devices such as dipsticks and/or strips. The FLISA is identical to the ELISA, except that the enzyme is replaced with a fluorescent dye. All immunochemical assays are based on the highly specific and sensitive binding between an antigen and an antibody. Thus, a monoclonal antibody against malaria parasite antigen was used to generate the bioconjugate with coumarin-derived dendrimer.

**Figure 6:**
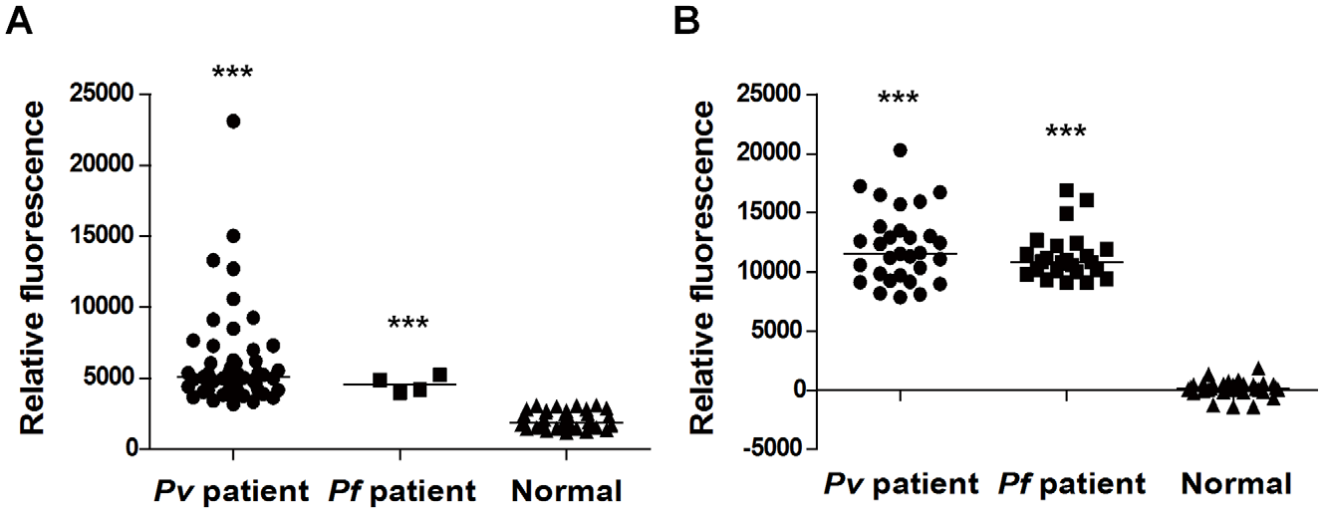
FLISA application of coumarin-derived dendrimer and mAb bioconjugates. FLISA results from two independent experiments are shown in graphs. Tests are conducted with blood samples from (A) normal (n = 34), patients infected with *P. vivax* (n = 50) and *P. falciparum* (n = 4) or (B) normal (n = 36), patients infected with *P. vivax* (n = 30) and *P. falciparum* (n = 24). Median is indicated. ***, *p*<0.0001 (Normal vs *Pv* or *Pf* patients).

Monoclonal antibodies against lactate dehydrogenase (LDH) of malaria parasites were efficiently and easily conjugated with the coumarin-derived dendrimer (see Supporting Protocol S1). Presence of malaria parasites in the blood samples tested was firstly confirmed by microscopic observation and nested PCR (see Supporting Protocol S1). Sandwich-FLISA was performed by coating antibody 1 onto a 96-well plate, applying the blood samples, reacting with antibody 2-coumarin-derived dendrimer bioconjugate, and detecting the reaction with a fluorescence microplate reader at a specific wavelength. Relative fluorescence is further determined among normal, *P. falciparum* and *P. vivax* infected blood samples (see Materials and Methods). As shown in Figure 6 and Table 1, FLISA was successfully able to detect *P. falciparum* and *P. vivax* infections in patient blood. Both *Plasmodium* infections were detected from two independent FLISA experiments leading to 100% sensitivity and specificity of assay (*p*<0.0001). Parasitemia of *P. vivax* infected samples ranged from 0.002% to 0.58% and the number of parasites per µl was ranging from 155 to 16,475. The number of parasites per µl of *P. falciparum* infected samples was from 39 to 48,863. Conventional ELISA method using a commercially available kit (see Supporting Protocol S1) was compared to FLISA. ELISA showed 90% sensitivity (data not shown), whereas FLISA using the novel coumarin-derived dendrimer resulted in 100% sensitivity and specificity for malaria detection. In order to further test the possibility of false positive in FLISA, we performed FLISA with clinical samples of HCV and HIV infection, which can be also detected in blood samples. The result demonstrated that FLISA system using the novel coumarin-derived dendrimer is very specific once bioconjugating monoclonal antibody is specific for antigen (Figure S1A and Table S1). These suggest that the novel coumarin-derived dendrimer is highly sensitive and applicable for immunochemical assay for disease diagnosis.

**Table 1:** Application of coumarin-derived dendrimer for FLISA-based malaria diagnosis.

Experiment #1		FLISA		Sum
		+	−	
Malaria parasite¶ examination	*Pv* +	50	0	50
	*Pf* +	4	0	4
	–	0	34	34
Sum		54	34	88
Sensitivity§		100% (54/54)		
Specificity		100% (34/34)		
**Experiment #2**		**FLISA**		**Sum**
		**+**	**–**	
Malaria parasite¶ examination	*Pv* +	30	0	30
	*Pf* +	24	0	24
	–	0	36	36
Sum		54	36	90
Sensitivity		100% (54/54)		
Specificity		100% (36/36)		

FLISA, fluorescence-linked immunosorbent assay; Pv: Plasmodium vivax; Pf: Plasmodium falciparum.

¶ The presence of malaria parasites was examined either by microscopy and nested PCR methods.

§ Conventional ELISA was performed using commercially available kit to compare sensitivity. The sensitivity and specificity were determined to 90% (49/54) and 100% (34/34), respectively.

### The Application of Coumarin-derived Dendrimer to Fluorescent Immunochromatographic Test (FICT) for Detecting Malaria Parasite

Fluorescent immunochromatographic test (FICT) is one of the most sensitive and promising approaches for quantifying low concentrations of target materials in biological samples. In addition, this assay system can be applied to develop simple, rapid, and sensitive miniaturized biomonitoring devices for point-of-care (POC) diagnosis [5]–[7].

**Figure 7:**
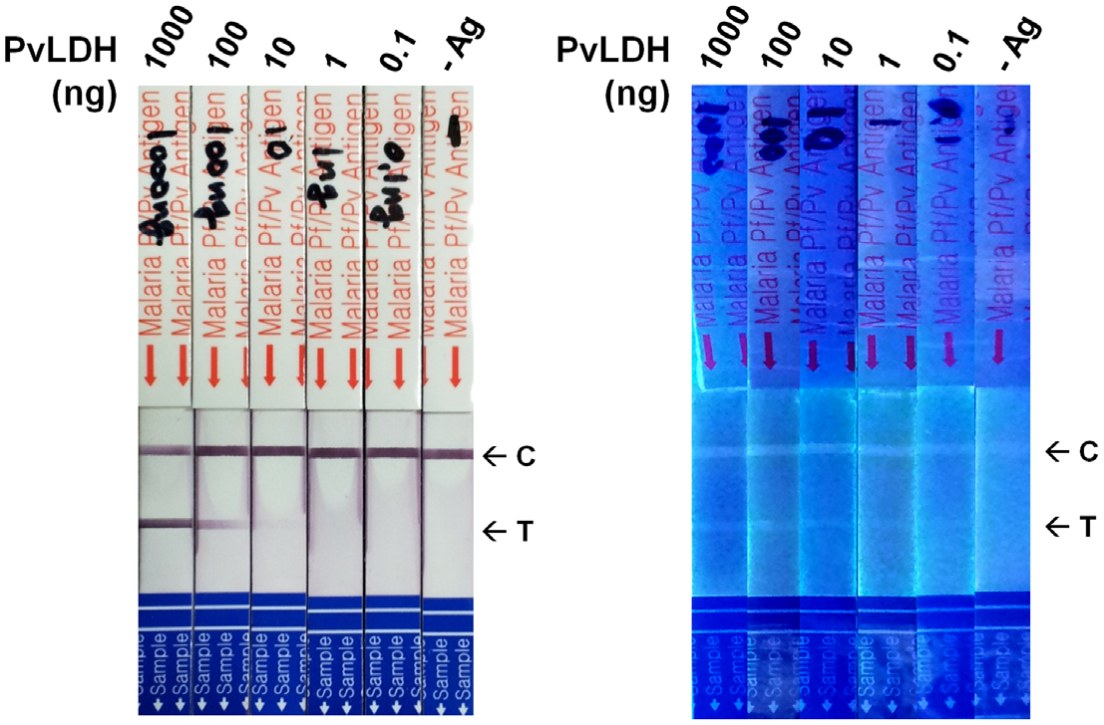
FICT application of coumarin-derived dendrimer and mAb bioconjugates. Recombinant *Pv*LDH as antigens are detected with anti-LDH mAb bioconjugate with colloidal gold (A) or coumarin-derived dendrimer (B). C indicates control line and T indicates test line for *P. vivax*. Test line is clearly shown at up to 0.1 ng antigen in coumarin-derived dendrimer bioconjugates but diminished to 10 ng antigen in colloidal gold bioconjugates.

In order to test the application of the novel coumarin-derived dendrimer to FICT in a strip format, a specifically modified approach with a commercially available strip test kit using colloidal gold conjugates of anti-*Plasmodium* LDH monoclonal antibodies was performed. The comparison of performance between the colloidal gold conjugates and the coumarin-derived dendrimer was necessary and important because colloidal gold is the reference for immunochromatographic testing (ICT) [66]. Here, the gold bioconjugates pad was removed and replaced with coumarin-derived dendrimer bioconjugates. The same monoclonal antibody used to generate the colloidal gold bioconjugates was labeled with coumarin-derived dendrimers. Purified recombinant *P. vivax* LDH proteins were used as antigens and serial dilutions of antigens were tested by ICT using the gold conjugates and FICT with the novel coumarin fluorophore. The colorimetric signal intensity of reaction with colloidal gold bioconjugates was qualitatively estimated by eye and the fluorescent signal intensity obtained with the coumarin fluorophore bioconjugates was measured using a Kodak imaging system with a 590 nm band pass filter. As shown in Figure 7, the signal by colloidal gold bioconjugates was clearly observed at antigen amount of 1,000 and 100 ng, but the signal became weaker at 10 ng. The fluorescent signal from the coumarin fluorophore bioconjugates was observed at an antigen amount of 0.1 ng, which was 100 times lower than the detection level with gold bioconjugates.

**Figure 8:**
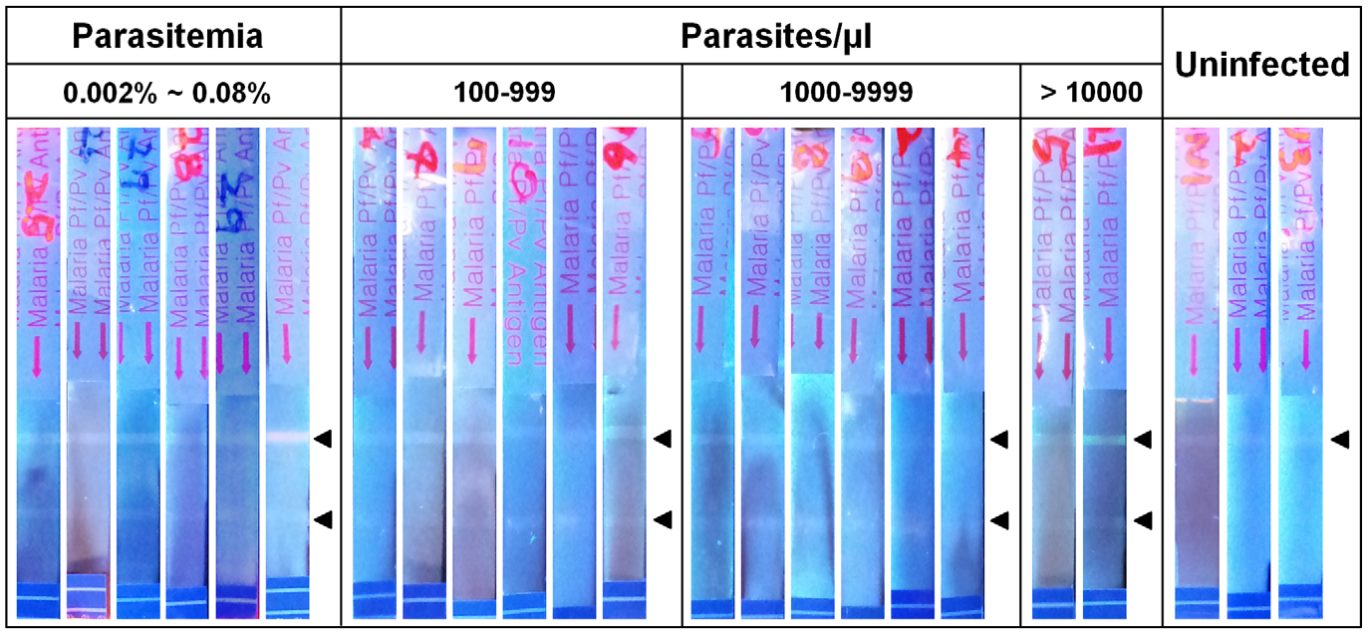
FICT-based diagnosis for *P. vivax* infection. Blood samples of *P. vivax* infected patients were categorized to several groups according to the level of parasitemia. Diagnostic results of *P. vivax* infected blood samples were compared to those of uninfected normal blood samples to determine positive or negative for infection. Upper arrow indicates control line and lower arrow indicates test line for *P. vivax*.

We further examined whether this FICT using novel coumarin-derived dendrimer is indeed applicable to diagnose *P. vivax* infection in humans. The similar test was conducted using blood samples of *P. vivax* infected patients as antigens instead of *P. vivax* LDH proteins. The dynamic range of sensitivity was assessed with the various levels of parasitemia. As shown in Figure 8, the fluorescent signals were apparent at test lines of all tested strips and those signal were enough to determine positive or negative for infection, even though the background noise was seen. FICT using clinical samples of HCV and HIV infection was further performed to test the possibility of false positive detection in the FICT system and it was found that the FICT system was highly specific (Figure S1B). These suggest that the novel coumarin-derived dendrimer paired with specific antibodies is extremely sensitive, specific and applicable for the development of a rapid and quantitative biosensor using FICT as a diagnostic method.

## Discussion

In this study, a novel fluorophore derived from coumarin with high fluorescence emission intensity, strong signal brightness, and high photostability has been developed. Coumarin was chosen because it is relatively easy to synthesize and thereby easy to modify to make a variety of derivatives. In addition, because of its relatively small size, it is suitable to generate dendrimer structures that produce superior fluorescence signals but do not alter the nature of biomolecules. Thus, the development of the coumarin-derived dendrimer in this study was also undertaken to improve the fluorescence intensity of coumarin derivatives developed previously [25]. The dendrimer emits strong and stable fluorescence at 512 nm upon excitation in a wide absorption band centered at 455 nm. The only emission spectrum of the fluorophore is shifted to 590 nm after simple bioconjugation with monoclonal antibody, resulting in large separation between the absorption and emission wavelengths. Bathochromic shift (i.e. a shift to longer wavelength) of fluorescent dyes is due to some reasons. Using solutions of different concentrations where there is considerable overlap of the fluorescence and absorption bands is one of reasons. In particular, the absorption energy and intensity decrease steadily upon increasing a conjugation. Substitution with an atom containing non-bonding electrons (auxochromic groups: -OH, OR, -NH_2_, -NHR, -SH, -SR, and -Hal) or conjugation of two or more double bonds can result in decreasing the energy difference between the HOMO (highest occupied molecular orbital) and LUMO (highest occupied molecular orbital) (i.e. energy level). However, it has been shown that a methyl group substituting different positions (a methyl group present either in the benzene or in the pyrone ring) in coumarin and chromone fails to cause any significant bathochromic shift of the main absorption bands [67]. This was considered to be due to the weak auxochromic property of the methyl group. Consistent with this report, the developed coumarin-derived dendrimer (see Supporting Protocol S1) did not exhibit any significant bathochromic shift of absorption band after its conjugation. A shift to longer emission wavelength (bathochromic shift) of coumarin-derived dendrimer after its conjugation to antibody is probably due to the substitution by -NH_2_ groups that are common compounds of antibody or any kind of protein. It is because the bathochromic wavelength shift of a fluorescent dye is usually about 60 nm for auxochromic groups and the shift in bioconjugated coumarin-derived dendrimer is observed to 70 nm.

The photostability is one of the most important requirements for an ideal fluorescent dye. There has been constant and continuous progress to improve photostability. A series of Alexa dye has been developed with similar absorption and emmistion spectra with various organic fluorescent dyes such as fluorescein, Texas Red, and rhodamine. Since Alexa dyes improved photointensity and photostability after their conjugations, they are commonly used and considered as the most effective organic fluorescent dyes for immunohistochemistry. However, considering the fluorescence intensity of Alexa dyes drops to 60% within 10 min after their conjugations to antibody, we believe the photostability is remarkably improved in our bioconjugate of novel coumarin-derived dendrimer. The bioconjugates of the coumarin-derived dendrimer were very stable within 10 min. The fluorescence intensity was maintained up to 80%. The spectroscopic properties of the coumarin-derived dendrimer, including the wide absorption band, the large separation between the absorption and emission wavelengths, and the high photostability, make it very suitable for use with LED-based diagnostic biosensors with minimized noise signal.

Finally, the capacity of the novel fluorophore for biosensing was tested by evaluating its efficacy in disease diagnosis by FLISA and FICT, which are the most desirable platforms for detection with diagnostic biosensors. FLISA using the novel fluorophore detected all *P. vivax* or *P. falciparum* infection leading to 100% sensitivity for malaria detection. Relative fluorescence was compared with control samples (No antigen added sample) to determine presence or absence of infection. The numerical value of fluorescence, especially the numerical mean value of fluorescence from normal samples, could be used to build an algorithm to determine presence or absence of infection in biosensor development. Further evaluation was carried out with ICT, and comparative analysis between colloidal gold and the novel fluorophore revealed that bioconjugates of coumarin-derived dendrimer detected antigens at concentrations 100 times lower than the colloidal gold bioconjugates. Furthermore, FICT results using *P. vivax* infected human bloods revealed that this novel coumarin-derived dendrimer is extremely sensitive and indeed applicable for the development of biosensor using FICT. The fluorescent signal was read by a UV imaging system that was not optimized to the spectral characteristics of the novel fluorophore. Thus, if a spectrum-optimized fluorometer can be engineered, the fluorescent signal of coumarin-derived dendrimer might be more apparent on the strip. Taken together, the spectral characteristics and clinical efficacy of the novel coumarin-derived dendrimer open, for the first time, the possibility of developing a cost/energy efficient LED-based portable quantitative biosensor for point-of-care (POC) disease diagnosis, which can provide for real-time monitoring (U-healthcare system) by a disease control center.

## Materials and Methods

### Ethics

Approval for the use of blood samples for this study was obtained from the Kangwon National University Hospital Institutional Review Board (Approval No. 10-041-07).

### Measurement of Spectroscopic Properties of Coumarin-derived Dendrimer

The absorption and emission spectra were measured by an ultraviolet/visible spectrometer (Spectra Academy, K-MAC, Korea) with a Czerny-Turner monochromator and a linear charge-coupled detector array operating at room temperature. The sample solution in the standard cuvette with a 10 mm path length was illuminated by the excitation light, and the absorption/emission spectra were subsequently measured by the spectrometer at room temperature. The integration time varied with the sample concentration. Deuterium/tungsten lamp was used for absorption measurements. The emission spectra were obtained from an ultraviolet laser at 375 nm or filtered broadband light whose peak wavelength matches with the absorption wavelength.

### Fluorescence-linked Immunosorbent Assay (FLISA)

Anti-*Pf*LDH monoclonal antibodies (5G6), purchased from C&K BioResource Inc. (South Korea), were prepared at 10 µg/ml concentration. 100 µl monoclonal antibodies were added to each well of a 96-well microtiter plate (Greiner, Germany) and incubated at 4°C overnight to immobilize the antibodies. The wells were washed 4 times with PBS-T (0.1% Tween-20 in 1X phosphate-buffered saline (PBS)) solution. The wells were blocked with 200 µl of 1% casein-based blocking buffer at 37°C for 2 h and then washed 4 times with PBS-T solution. *P. falciparum* and *P. vivax* infected blood samples were added at 1∶100 dilution in PBS and incubated at 27°C for 1 h. Excess antigens were washed 5 times with PBS-T solution. The synthesized bioconjugates of novel fluorophore-anti-*Pf*LDH monoclonal antibodies (8C10) were added and incubated at 27°C for 1 h. Stringent washing was performed 6 times to remove unbound proteins and 100 µl PBS was added to each well. The specific antigen-antibody binding was determined by measuring fluorescence with 460 nm excitation (Bandwidth 20 nm) and 560 nm emission (Bandwidth 20 nm) filters using an Infinite F200 microplate reader (TECAN, Switzerland). Relative fluorescence was determined by extracting fluorescence value of control (No blood added sample) from those of normal, *P. falciparum* and *P. vivax* infected blood samples. A sample showing higher fluorescence value than mean fluorescence value ± SD of normal samples was considered to be positive. Student’s *t*-test was performed between normal and *Pv* or *Pf* infected patient groups to determine statistical significance.

### Fluorescent Immunochromatographic Test (FICT)

Anti-*Pf*LDH monoclonal antibody (8C10) and recombinant *Pv*LDH protein were purchased from C&K BioResource Inc. (South Korea). ASAN Easy Test Malaria Pf/Pv Strip (Asan Pharmaceutical Co. Ltd., South Korea) was used for the modified test of coumarin-derived dendrimer bioconjugates. The gold bioconjugates pad was removed and replaced with a pad containing coumarin-derived dendrimer bioconjugates. Purified recombinant *P. vivax* LDH proteins were serially diluted from 1,000 ng/µl to 0.1 ng/µl concentrations and each 1 µl recombinant protein solution was applied to strips. For clinical test, 5 µl blood of *P. vivax* infected patients was applied to strips. The strip test was performed according to the manufacturer’s instruction. The colorimetric signal of colloidal gold was qualitatively read with the naked eye in 15 m. The fluorescent signal obtained with the coumarin-derived dendrimer was measured using a Kodak Gel Logic 100 imaging system, which has a 590 nm band pass filter (Carestream Molecular Imaging, USA). The test reaction was conducted for 15 m and the results were determined within another 15 m.

## Supporting Information

Figure S1 Test for the possibility of false positive detection in the FICT system. (A) FLISA are conducted with blood samples from normal (n = 20), patients infected with *P. vivax* (n = 10), patients infected with HCV (n = 20) and patients infected with HIV (n = 5). (B) FICT-based diagnostic results of blood samples were compared. Upper arrow indicates control line. Middle and lower arrows indicate test lines for *P. vivax* and *P. falciparum* infection, respectively.
(TIF)

Table S1 Test for cross-reactivity of FLISA for malaria diagnosis. HCV: Hepatitis C Virus; HIV: Human immunodeficiency virus.
(DOCX)

Supporting Protocol S1 (DOCX)
