# Supplementary figures and images for: Development of a Novel Fluorophore for Real-Time Biomonitoring System
Source: PLoS One. 2012 Nov 2;7(11):e48459. doi: 10.1371/journal.pone.0048459 (PMC3487730; doi:10.1371/journal.pone.0048459)

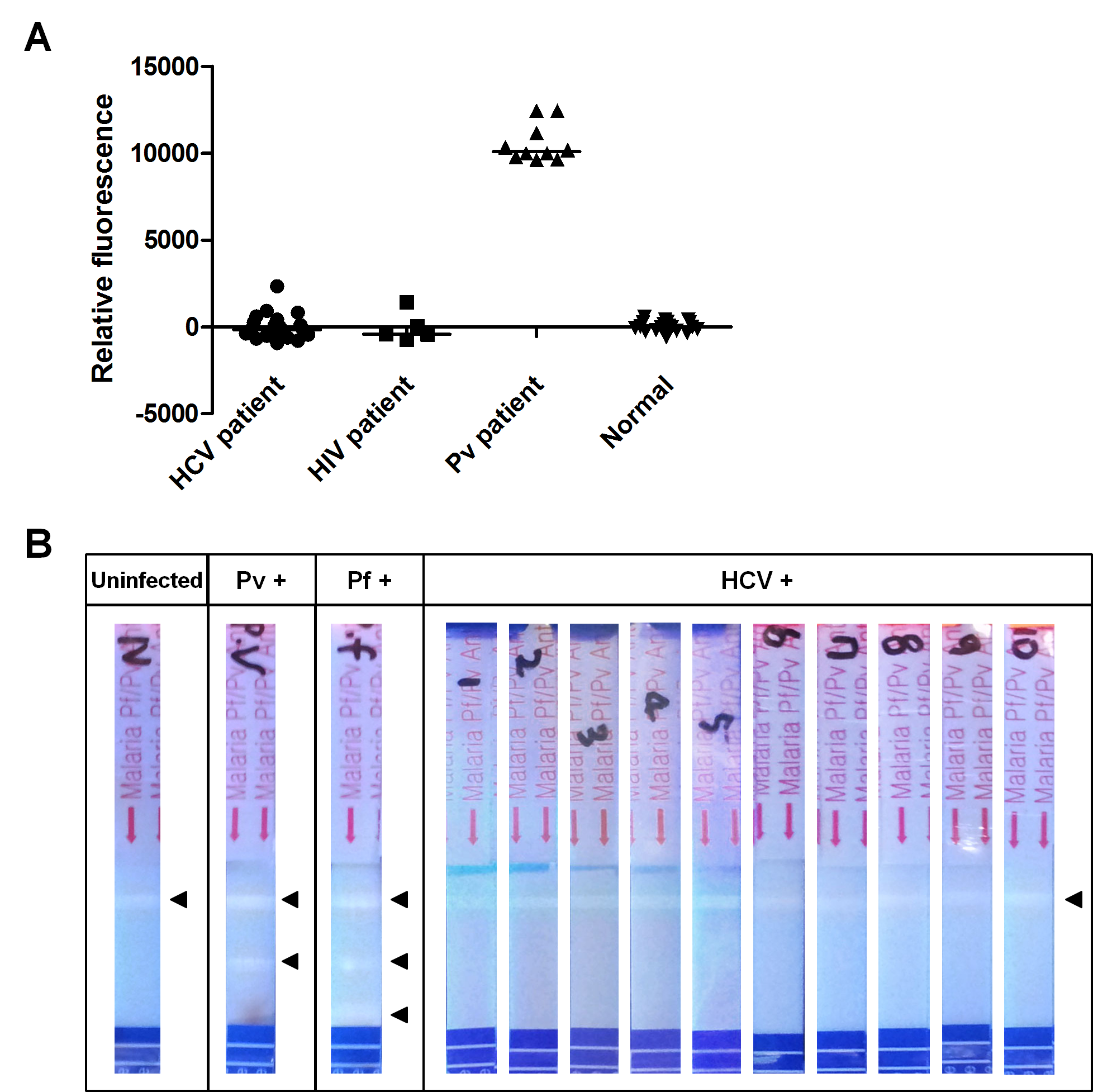

Supplement: Figure S1 — Test for the possibility of false positive detection in the FICT system. (A) FLISA are conducted with blood samples from normal (n = 20), patients infected with P. vivax (n = 10), patients infected with HCV (n = 20) and patients infected with HIV (n = 5). (B) FICT-based diagnostic results of blood samples were compared. Upper arrow indicates control line. Middle and lower arrows indicate test lines for P. vivax and P. falciparum infection, respectively. (TIF) [file pone.0048459.s001.tif]
