# Supplementary material for: Development of a Novel Fluorophore for Real-Time Biomonitoring System
Source: PLoS One. 2012 Nov 2;7(11):e48459. doi: 10.1371/journal.pone.0048459 (PMC3487730; doi:10.1371/journal.pone.0048459)
Supplement: Table S1 — Test for cross-reactivity of FLISA for malaria diagnosis. HCV: Hepatitis C Virus; HIV: Human immunodeficiency virus. (DOCX) [file pone.0048459.s002.docx]

Table S1. Test for cross-reactivity of FLISA for malaria diagnosis

| Experiment #1 | | FLISA | | | Sum |
| --- | --- | --- | --- | --- | --- |
|  |  | + | | - |  |
| *Plasmodium* + | Pv | 10 | 0 | | 10 |
| *Plasmodium* - | HCV | 0 | 20 | | 20 |
|  | HIV | 0 | 5 | | 5 |
|  | Uninfected | 0 | 20 | | 20 |
| Sum | | 10 | 45 | | 55 |
| Sensitivity | | 100% (10/10) | | | |
| Specificity | | 100% (45/45) | | | |

HCV: Hepatitis C Virus; HIV: Human immunodeficiency virus
