# Supplementary material for: Development of a Novel Fluorophore for Real-Time Biomonitoring System
Source: PLoS One. 2012 Nov 2;7(11):e48459. doi: 10.1371/journal.pone.0048459 (PMC3487730; doi:10.1371/journal.pone.0048459)
Supplement: Supporting Protocol S1 — (DOCX) [file pone.0048459.s003.docx]

**Supporting Protocol**

**Synthesis of 3-(isopropylamino)phenol 1**

To a stirred suspension of sodium borohydride (2.70 g, 71.37 mmol) in THF (50 mL), at 0 ^o^C, was added glacial acetic acid (12.46 ml, 115.3 mmol) and stirred for 1 h, then reaction mixture was brought to room temperature and stirred overnight. To the above stirred solution was successively added 3-aminophenol (2.0 g, 18.32 mmol) and acetone (13.42 mL, 183.2 mmol) and stirred for 12 h. The reaction mixture was washed with saturated sodium bicarbonate solution (30 mL), water (30 mL) and brine (20 mL) respectively, organic layer was dried over anhydrous sodium sulfate and concentrated *in vacuo* and purified by column chromatography using hexane and ethyl acetate (4:1) to give 3-(isopropylamino)phenol **1** (2.16 g, 78%). ^1^H NMR (500 MHz, CDCl_3_) δ 6.99 (t, 1H, *J* = 8.25 Hz), 6.12-6.17 (m, 2H), 6.07 (t, 1H, *J* = 2.3 Hz), 3.54-3.61 (m, 1H), 1.19 (d, 6H, *J* = 6.4Hz). ^13^C NMR (125 MHz, CDCl_3_) δ 156.7, 149.1, 130.2, 106.5, 103.9, 99.9, 44.3, 23.0 (2).

**Synthesis of 3-(isopropyl(prop-2-ynyl)phenol 2**

To a stirred solution of 3-(isopropylamino)phenol (2.0 g, 13.22 mmol) in toluene (15 mL) was successively added Hunig’s base (5.75 mL, 33.05 mmol) and propargyl chloride (2.36 mL, 33.05 mmol). The reaction mixture was stirred for 10 h at 50^o^C and allowed to cool to room temperature. The residue was partitioned between ethyl acetate (30 mL) and water (30 mL). The separated organic layer was washed with brine (10mL), dried over anhydrous sodium sulfate, concentrated *in vacuo* and purified by column chromatography using hexane and ethyl acetate (6:1) to give 3-(isopropyl(prop-2-ynyl)phenol **2** (2.22 g, 89%). ^1^H NMR (500 MHz, CDCl_3_) δ 7.03 (t, 1H, *J* = 8.2 Hz), 6.40 (dd, 1H, *J* = 8.2Hz), 6.32 (s, 1H), 6.16 (dd, 1H, *J* = 7.7Hz), 3.98-4.07 (m, 1H), 3.85 (d, 2H, *J* = 2.2Hz), 2.10 (t, 1H, *J* = 2.3Hz), 1.18 (d, 6H, *J* = 6.9Hz). ^13^C NMR (125 MHz, CDCl_3_) δ 156.7, 149.8, 130.1, 106.4, 104.3, 100.7, 82.2, 71.1, 48.8, 33.6, 20.1 (2).

**Synthesis of 2-hydroxy-4-(isopropyl(prop-2-ynyl)amino)benzaldehyde 3**

To stirred DMF (13.30 mL, 172.6 mmol), at 0 ^o^C, was added POCl_3_ (2.68 mL, 28.79 mmol) dropwise and the reaction mixture was stirred for 20 min, and brought to room temperature and stirred for 1 h. To the above stirred solution, 3-(isopropyl(prop-2-ynyl)phenol (2.18 g, 11.51 mmol) dissolved in DMF (10 mL) was added and stirred for 12 h. The reaction mixture was diluted with water (30 mL) and neutralized by sodium bicarbonate. The residue was partitioned between ethyl acetate (30 mL) and water. The separated organic layer was washed with brine (10 mL), dried over anhydrous sodium sulfate, concentrated *in vacuo* and purified by column chromatography using hexane and ethyl acetate (7:1) to give 2-hydroxy-4-(isopropyl(prop-2-ynyl)amino)benzaldehyde **3** (2.05 g, 82%). ^1^H NMR (500 MHz, CDCl_3_) δ 11.52 (s, 1H), 9.55 (s, 1H),7.33 (d, 1H, *J* = 8.7 Hz), 6.46 (dd, 1H, *J* = 2.3, 8.7Hz), 6.29 (d, 1H, *J* = 2.3Hz), 4.19-4.22 (m, 1H), 3.98 (d, 2H, *J* = 2.7Hz), 2.23 (t, 1H, *J* = 2.7Hz), 1.29 (d, 6H, *J* = 6.9Hz). ^13^C NMR (125 MHz, CDCl_3_) δ 192.7, 164.2, 154.7, 135.3, 112.4, 105.4, 98.5, 80.4, 72.0, 49.1, 33.3, 20.2 (2).

**Synthesis of 3-(benzo[d]thiazol-2-yl)-7-isopropyl(prop-2-ynyl)amino)-2H-chromen-2-one 4**

To a stirred solution of 2-hydroxy-4-(isopropyl(prop-2-ynyl)amino)benzaldehyde (1.8 g, 8.28 mmol) in ethanol (15 mL) was successively added ethyl 2-(benzo[d]thiazol-2-yl)acetate (1.83 g, 8.28 mmol) and piperidine (1.63 mL, 16.56 mmol). The reaction mixture was refluxed for 2 h and filtered to give the residue 3-(benzo[d]thiazol-2-yl)-7-isopropyl(prop-2-ynyl)amino)-2H-chromen-2-one **4** (1.54 g, 50%). ^1^H NMR (500 MHz, CDCl_3_) δ 8.94 (s, 1H), 8.02 (d, 1H, *J* = 8.2 Hz),7.94 (d, 1H, *J* = 7.8 Hz), 7.54 (d, 1H, *J* = 8.7Hz), 7.47-7.50 (m, 1H), 7.35-7.38 (m, 1H), 6.88 (dd, 1H, *J* = 2.3, 8.7Hz), 6.80 (d, 1H, *J* = 2.3Hz), 4.21-4.26 (m, 1H), 4.04 (d, 2H, *J* = 2.3Hz), 2.26 (t, 1H, *J* = 2.2Hz), 1.34 (d, 6H, *J* = 6.4Hz). ^13^C NMR (125 MHz, CDCl_3_) δ 161.5, 161.0, 156.7, 152.6, 152.4, 142.0, 136.4, 130.6, 126.2, 124.7, 122.3, 121.7, 113.9, 111.0, 109.8, 98.8, 80.0, 72.3, 49.5, 33.5, 20.2 (2).

**Synthesis of 2-(2-(2-azidoethoxy)ethoxy)ethanol 5**

To a stirred solution of 2-(2-(2-chloroethoxy)ethoxy)ethanol (3.48 g, 20.63 mmol) in DMF (15 mL), at 100 ^o^C, was added NaN_3_ (4.02 g, 61.89 mmol). The reaction mixture was stirred for 12 h. After the DMF was evaporated, residue was partitioned between water (40 mL) and ethyl acetate (40 mL). The separated organic layer was washed with the brine (15 mL), dried over anhydrous sodium sulfate, concentrated *in vacuo* and purified by column chromatography using hexane and ethyl acetate (2:1) to give 2-(2-(2-azidoethoxy)ethoxy)ethanol **5** (3.05 g, 84%). ^1^H NMR (500 MHz, CDCl_3_) δ 3.72-3.74 (m, 2H), 3.65-3.69 (m, 6H), 3.60-3.62 (m, 2H), 3.39 (t, 2H, *J* = 5.0 Hz).

**Synthesis of 2-(2-(2-azidoethoxy)ethoxy)ethyl 4-methylbenzenesulfonate 6**

To a stirred solution of 2-(2-(2-azidoethoxy)ethoxy)ethanol (3.05 g, 17.40 mmol) in DCM (15 mL) was successively added DMAP (212.6 mg, 1.74 mmol), TEA (7.28 mL, 52.20 mmol) and tosyl chloride (3.98 g, 20.88 mmol). The reaction mixture was stirred for 12 h and residue was partitioned between water (40 mL) and ethyl acetate (60 mL). The separated organic layer was washed with the brine (15 mL), dried over anhydrous sodium sulfate and concentrated *in vacuo* to give 2-(2-(2-azidoethoxy)ethoxy)ethyl 4-methylbenzenesulfonate **6** (5.15 g, 90%). ^1^H NMR (500 MHz, CDCl_3_) δ 7.79 (d, 2H, *J* = 8.2Hz), 7.33 (d, 2H, *J* = 8.2Hz), 4.15 (t, 2H, *J* = 5.0 Hz), 3.69 (t, 2H, *J* = 5.0 Hz), 3.63 (t, 2H, *J* = 5.5 Hz), 3.59 (s, 4H), 3.36 (t, 2H, *J* = 5.0 Hz), 2.44 (s, 3H). ^13^C NMR (125 MHz, CDCl_3_) δ 144.9, 133.0, 129.9 (2), 128.0 (2), 70.8, 70.7, 70.1, 69.3, 68.8, 50.7, 21.7.

**Synthesis of methyl gallate 7**

To a stirred solution of gallic acid (5.0 g, 29.39 mmol) in methanol (25 mL) at room temperature was successively added conc. H_2_SO_4_ (0.5 mL) and trimethylorthoformate (3.21 mL, 88.17 mmol). The reaction mixture was refluxed for 10 h and allowed to cool to room temperature. After methanol was evaporated, the water was added to the mixture and filtered to give the residue methyl gallate**7** (5.15 g, 95%). ^1^H NMR (500 MHz, DMSO-D_6_) δ 9.29 (s, 2H), 8.96 (s, 1H), 6.92 (s, 2H), 3.73 (s, 3H).

**Synthesis of methyl 3,4,5-tris(2-(2-(2-azidoethoxy)ethoxy)ethoxy)benzoate** **8**

To a stirred solution of 2-(2-(2-azidoethoxy)ethoxy)ethyl 4-methylbenzenesulfonate (4.80 g, 14.57 mmol) in acetone (15 mL) was successively added K_2_CO_3_ (2.05 g, 14.85 mmol), methyl gallate (650.9 mg, 3.53 mmol) and tetrabutylammonium bromide (25 mg). The reaction mixture was refluxed for 17 h and residue was partitioned between water (40 mL) and ethyl acetate (60 mL). The separated organic layer was washed with brine (15 mL), dried over anhydrous sodium sulfate, concentrated *in vacuo* and purified by column chromatography using hexane and ethyl acetate (1:1.5) to give methyl 3,4,5-tris(2-(2-(2 azidoethoxy)ethoxy)ethoxy)benzoate **8** (2.18 g, 94%). ^1^H NMR (500 MHz, CDCl_3_) δ 7.29 (s, 2H), 4.18-4.23 (m, 6H), 3.86-3.88 (m, 7H), 3.81 (t, 2H, *J* = 5.0 Hz), 3.70-3.74 (m, 6H), 3.63-3.67 (m, 12H), 3.36-3.39 (m, 6H).

**Synthesis of 3,4,5-tris(2-(2-(2-azidoethoxy)ethoxy)ethoxy)benzoic acid 9**

To a stirred solution of methyl 3,4,5-tris(2-(2-(2-azidoethoxy)ethoxy)ethoxy) benzoate (2.18 g, 3.32 mmol) in THF (15 mL) was added aq. LiOH (1 M) (9.96 mL, 9.96 mmol). The reaction mixture was refluxed for 4 h, allowed to cool to room temperature and 0.1 N HCl was added till pH 4. The residue was partitioned between water (40 mL) and ethyl acetate (40 mL). The separated organic layer was washed with brine (15 mL), dried over anhydrous sodium sulfate and concentrated *in vacuo* to give 3,4,5-tris(2-(2-(2-azidoethoxy)ethoxy)ethoxy) benzoic acid **9** (2.90 g, 98%). ^1^H NMR (500 MHz, CDCl_3_) δ 7.35 (s, 2H), 4.18-4.29 (m, 6H), 3.81-3.88 (m, 6H), 3.73 (m, 6H), 3.65-3.68 (m, 12H), 3.38 (m, 6H). ^13^C NMR (125 MHz, CDCl_3_) δ 170.9, 152.4, 143.3, 124.0, 109.6, 72.5, 70.9 (3), 70.8 (3), 70.6, 70.1 (3), 69.8 (2), 68.9 (2), 50.7 (3).

**Synthesis of methyl 4-(aminomethyl)benzoate 10**

A mixture of 4-(aminomethyl)benzoic acid (1.0 g, 6.61 mmol) and chlorotrimethylsilane (3.35 mL, 26.44 mmol) was stirred for 30 minutes and added anhydrous methanol (20 mL). The reaction mixture was stirred for 48 h and concentrated *in vacuo* to give methyl 4-(aminomethyl)benzoate **10** (1.25 g, 94%). ^1^H NMR (500 MHz, D_2_O) δ 8.02 (d, 2H, *J* = 6.4 Hz), 7.51 (d, 2H, *J* = 8.2 Hz), 4.20 (s, 2H), 3.88 (s, 3H).

**Synthesis of methyl 4-((3,4,5-tris(2-(2-(2-azidoethoxy)ethoxy)ethoxy) benzamido)methyl) benzoate 11**

To a stirred solution of 3,4,5-tris(2-(2-(2-azidoethoxy)ethoxy)ethoxy)benzoic acid (1.29 g, 2.01 mmol) in DCM (10 mL) was successively added methyl 4-(aminomethyl)benzoate (810 mg, 4.02 mmol), TEA (1.17 mL, 8.4 mmol) and EDAC (770.6 mg, 4.02 mmol). The reaction mixture was stirred at room temperature for 36 h, then diluted with ethyl acetate (30 mL) and successively washed with 0.5 N HCl (20 mL), water (20 mL), and brine (10 mL), dried over anhydrous sodium sulfate, concentrated *in vacuo* and purified by column chromatography using hexane, ethyl acetate and methanol (4:4:1) to give methyl 4-((3,4,5-tris(2-(2-(2-azidoethoxy)ethoxy)ethoxy)benzamido)methyl)benzoate **11** (941 mg, 60%). ^1^H NMR (500 MHz, CDCl_3_) δ 8.01 (d, 2H, *J* = 8.2Hz), 7.40 (d, 2H, *J* = 8.2Hz), 7.08 (s, 2H), 6.57 (bs, 1H), 4.67 (d, 2H, *J* = 5.5Hz), 4.20 (m, 6H), 3.90 (s, 3H), 3.84 (m, 4H), 3.80 (t, 2H, *J* = 5.0Hz), 3.68-3.71 (m, 6H), 3.63-3.65 (m, 12 H), 3.33-3.38 (m, 6H). ^13^C NMR (125 MHz, CDCl_3_) δ 166.9 (2), 152.6 (2), 143.6, 141.8, 130.1 (2), 129.4, 129.3, 127.7 (2), 107.3 (2), 72.4, 70.8 (3), 70.7 (3), 70.6, 70.1, 70.0 (2), 69.8 (2), 69.2 (2), 52.2, 50.7 (3), 43.8.

**Synthesis of 4-((3,4,5-tris(2-(2-(2-azidoethoxy)ethoxy)ethoxy)benzamido) methyl)benzoic acid 12**

To a stirred solution of methyl 4-((3,4,5-tris(2-(2-(2-azidoethoxy)ethoxy) ethoxy)benzamido)methyl)benzoate (930 mg, 1.17 mmol) in THF (10 mL) was added aq. LiOH (1 M) (7.02 mL, 7.02 mmol). The reaction mixture was stirred at room temperature for 4 h, allowed to cool to room temperature and 0.1 N HCl was added till pH 4. The residue was partitioned between water (30 mL) and ethyl acetate (40 mL). The separated organic layer was washed with brine (15 mL), dried over anhydrous sodium sulfate and concentrated *in vacuo* to give 4-((3,4,5-tris(2-(2-(2-azidoethoxy)ethoxy)ethoxy)benzamido)methyl)benzoic acid **12** (897 mg, 99%). ^1^H NMR (500 MHz, CDCl_3_) δ 8.04 (d, 2H, *J* = 8.2Hz), 7.42 (d, 2H, *J* = 8.2Hz), 7.10 (s, 2H), 6.65 (bs, 1H), 4.68 (d, 2H, *J* = 4.6Hz), 4.22 (m, 6H), 3.85 (t, 4H, *J* = 4.6Hz), 3.80 (t, 2H, *J* = 5.0Hz), 3.70-3.71 (m, 6H), 3.63-3.66 (m, 12 H), 3.33-3.38 (m, 6H).

^13^C NMR (125 MHz, CDCl_3_) δ 170.3, 167.1 152.6 (2), 144.4, 141.8, 130.6 (2), 129.2, 128.6, 127.7 (2), 107.4 (2), 72.4, 70.8 (3), 70.7 (3), 70.6, 70.1, 70.0 (2), 69.8 (2), 69.2 (2), 50.7 (3), 43.8.

**Synthesis of 4-methoxy benzyl 4-((3,4,5-tris(2-(2-(2-azidoethoxy)ethoxy) ethoxy)benzamido) methyl)benzoate 13**

To a stirred solution of 4-((3,4,5-tris(2-(2-(2-azidoethoxy)ethoxy)ethoxy) benzamido)methyl) benzoic acid (800 mg, 1.03 mmol) in DMF (8 mL) was successively added para-methoxy benzyl bromide (0.22 mL, 1.54 mmol) and sodium bicarbonate (346.1 mg, 4.12 mmol). The reaction mixture was stirred for 10 h. The residue was partitioned between water (30 mL) and ethyl acetate (30 mL). The separated organic layer was washed with brine (10 mL), dried over anhydrous sodium sulfate, concentrated *in vacuo* and purified by column chromatography using hexane, ethyl acetate and methanol (6:6:1) to give 4-methoxy benzyl 4-((3,4,5-tris(2-(2-(2-azidoethoxy)ethoxy)ethoxy)benzamido) methyl)benzoate **13** (755.8 mg, 82%). ^1^H NMR (500 MHz, CDCl_3_) δ 8.02 (d, 2H, *J* = 8.7Hz), 7.37 (t, 4H, *J* = 8.2Hz), 7.07 (s, 2H), 6.90 (m, 2H), 6.59 (t, 1H, *J* = 5.9Hz), 5.28 (s, 2H ), 4.65 (d, 2H, *J* = 5.9Hz), 4.19 (t, 6H, *J* = 5.0Hz), 3.84 (t, 4H, *J* = 5.0Hz), 3.80 (s, 3H), 3.79 (m, 2H), 3.69-3.71 (m, 6H), 3.62-3.65 (m, 12 H), 3.32-3.37 (m, 6H). ^13^C NMR (125 MHz, CDCl_3_) δ 166.9, 166.3, 159.7, 152.6 (2), 143.7, 141.7, 130.2 (2), 130.1 (2), 129.5, 129.3, 128.1, 127.7 (2), 114.0 (2), 107.3 (2), 72.4, 70.8 (3), 70.7 (3), 70.6, 70.1, 70.0 (2), 69.8 (2), 69.2 (2), 66.6, 55.3, 50.7 (3), 43.8.

**Synthesis of 4-methoxybenzyl4-((3,4,5-tris(2-(2-((4-(((3-(benzo[d]thiazol-2-yl)-2-oxo-2H-chromen-7-yl)(isopropyl)amino)methyl)-1H-1,2,3-triazol-1-yl)methoxy)ethoxy)ethoxy)benzami do)methyl)benzoate 14**

To a stirred solution of compound **4** (164.7 mg, 0.44 mmol) and compound **13** (100 mg, 0.11 mmol) in DCM (2 mL) and water (1 mL) was successively added CuSO_4_.5H_2_O (4.2 mg, 15 mol%) and sodium ascorbate (9.9 mg, 45 mol%). The reaction mixture was stirred for 8 h. The residue was partitioned between water (30 mL) and DCM (30 mL). The separated organic layer was washed with brine (10 mL), dried over anhydrous sodium sulfate, concentrated *in vacuo* and purified by column chromatography using chloroform and methanol (20:1) to give 4-methoxybenzyl4-((3,4,5-tris(2-(2-((4-(((3-(benzo[d]thiazol-2-yl)-2-oxo-2H-chromen-7-yl)(isopropyl)amino)methyl)-1H-1,2,3-triazol-1-yl)methoxy)ethoxy)ethoxy)benzamido)methyl)benzoate **14** ( 130.4 mg, 60%). ^1^H NMR (500 MHz), CDCl_3_) δ 9.17 (s, 3H), 8.06 (d, 2H, *J* = 7.3Hz), 7.86 (d, 3H, *J* = 7.8Hz), 7.76 (d, 3H, *J* = 7.7Hz), 7.48 (m, 4H), 7.35-7.39 (m, 9H), 7.30 (d, 3H, *J* = 7.8Hz), 7.18 (s, 2H), 6.89 (d, 2H, *J* = 8.7Hz), 6.71 (d, 3H, *J* = 7.8Hz), 6.58 (s, 3H), 5.22 (s, 2H), 4.47-4.61 (m, 14H), 4.23 (m, 3H), 4.01-4.03 (m, 6H), 3.79 (m, 9H), 3.62 (m, 6H), 3.48-3.52 (m, 12H), 1.25 (d, 18H, *J* = 6.4 Hz).

**Synthesis of 4-((3,4,5-tris(2-(2-((4-(((3-(benzo[d]thiazol-2-yl)-2-oxo-2H-chromen-7-yl)(isopropyl)amino)methyl)-1H-1,2,3-triazol-1-yl)methoxy)ethoxy) ethoxy)benzamido)methyl)benzoic acid 15**

To a stirred solution of compound **14** (120 mg, 0.06 mmol) in DCM (2 mL) was added TFA (1 mL). The reaction mixture was stirred at room temperature for 36 h. The reaction mixture was evaporated by adding ethanol (10 mL) for 6 times and concentrated *in vacuo* to give the residue **15** (105.7 mg, 95%).

**Synthesis of 2,5-dioxopyrrolidin-1-yl 4-((3,4,5-tris(2-(2-((4-(((3-(benzo[d] thiazol-2-yl)-2-oxo-2H-chromen-7-yl)(isopropyl)amino)methyl)-1H-1,2,3-triazol-1-yl)methoxy)ethoxy)ethoxy)benz amido)methyl)benzoate 16**

To a stirred solution of compound **15** (100 mg, 0.053 mmol) in DCM (2 mL) at 0^o^C was successively added N-hydroxysuccinimide (243.9 mg, 2.12 mmol) and EDAC (406.4 mg, 2.12 mmol). The reaction mixture was stirred for overnight. The residue was partitioned between DCM (20 mL) and water (20 mL). The separated organic layer was washed with brine (10 mL), dried over anhydrous sodium sulfate, concentrated *in vacuo* and purified by column chromatography using chloroform and methanol (20:1) to give 2,5-dioxopyrrolidin-1-yl 4-((3,4,5-tris(2-(2-((4-(((3-(benzo[d]thiazol-2-yl)-2-oxo-2H-chromen-7yl)(isopropyl)amino)methyl)-1H-1,2,3-triazol-1-yl)methoxy)ethoxy)ethoxy)benzamido)methyl) benzoate **16** (96 mg, 92%).

^1^H NMR (500 MHz, CDCl_3_) δ 10.04 (s, 3H), 8.32 (d, 2H, *J* = 8.2Hz), 8.11 (d, 3H, *J* = 9.6Hz), 7.93 (d, 4H, *J* = 7.3Hz), 7.74 (d, 3H, *J* = 7.3Hz), 7.45-7.60 (m, 9H), 7.37 (m, 3H), 6.82 (m, 3H), 6.65 (s, 3H), 4.53-4.70 (m, 14H), 4.34 (m, 3H), 4.01-4.13 (m, 6H), 3.82 (m, 9H), 3.65 (m, 6H), 3.52 (m, 12H), 2.91 (s, 4H), 1.25 (d, 18H, *J* = 6.4 Hz). ^13^C NMR (125 MHz, CDCl_3_) δ 169.3 (2), 167.0, 161.6, 161.3 (3), 160.9 (3), 156.6 (3), 153.0 (3), 152.5 (3), 152.3 (2), 146.6 (3), 145.6 (3), 142.0 (2), 141.9, 141.0 ,136.2 (3), 130.7 (2), 130.6, 129.4, 128.3 (2), 126.3 (3), 124.7 (3), 123.9, 123.2 (3), 122.2 (3), 121.6 (3), 113.2 (3), 111.2 (3), 109.4 (3), 107.1 (2), 98.3 (3), 72.3, 70.6 (3), 70.5 (3), 70.4, 69.5 (2), 69.4 (3), 68.6 (2), 50.4 (2), 50.3 (1), 49.3 (3), 43.7, 40.6 (3), 25.7 (2), 20.2 (6).

**Simple and efficient conjugation of coumarin derivative with antibodies**

The anti-*Pf*LDH monoclonal antibody (8C10) was purchased from C&K BioResource Inc. (South Korea). Monoclonal antibodies (mAbs) were first mixed with 0.1 M sodium bicarbonate (NaHCO_3_) solution (pH8.5) at a 1:1 ration (v/v) for 4 h at room temperature (RT) and incubated with coumarin-derived dendrimers with 6 moles of fluorophore per mole of antibody for overnight at 4 °C. Bovine serum albumin (BSA) was added to the fluorophore-mAb bioconjugates up to 1% and gently mixed for 2 h at RT. The synthesized bioconjugates were further dialyzed with phosphate buffered saline (PBS) solution to remove uncoupled coumarin-derived dendrimers and/or mAbs. The purified fluorophore-mAb bioconjugates were then used for FLISA and FICT.

**Confirmation of blood samples for the infection of malaria parasites**

Blood samples from *P. vivax*- and *P. falciparum*-infected patients were confirmed by microscopic observation, the gold standard method using light microscopy to detect and quantify malaria parasites. Blood sample was collected from patient’s finger and prepared with thin or thick blood smear on slide glass. The smear was then stained with Giemsa and examined under a light microscopy [1]. Nested PCR was used to further confirm the infection and performed according to the protocol indicated in the guideline of malaria diagnosis from Korean CDC, 2009. Conventional ELISA was conducted to compare the results of diagnosis. pLDH antigen of *Plasmodium* species was detected using ELISA kit according to manufacturer’s instruction (SD Malaria antigen ELISA kit, Standard Diagnostics, Inc., South Korea).

**Supporting Reference**

1. Payne D (1988) Use and limitations of light microscopy for diagnosing malaria at the primary health care level. Bull World Health Organ 66: 621-626.
